# The Effect of Diacerein on Type 2 Diabetic Mellitus: A Systematic Review and Meta-Analysis of Randomized Controlled Trials with Trial Sequential Analysis

**DOI:** 10.1155/2020/2593792

**Published:** 2020-02-10

**Authors:** Shizhe Guo, Xianshan Guo, Hongya Zhang, Xuan'e Zhang, Zhen Li

**Affiliations:** ^1^Internal Medicine Base, Yangpu Hospital, Tongji University School of Medicine, Shanghai 200090, China; ^2^Department of Endocrinology, Xinxiang Central Hospital, Xinxiang, Henan 453000, China; ^3^Central Laboratory, Yang Pu District Center of Disease Control and Prevention, Shanghai 200090, China; ^4^Department of Endocrinology, Yangpu Hospital, Tongji University School of Medicine, Shanghai 200090, China; ^5^Department of General Surgery, Yangpu Hospital, Tongji University School of Medicine, Shanghai 200090, China

## Abstract

**Aims:**

To figure out the effect of diacerein supplementation on type 2 diabetes mellitus (T2DM).

**Methods:**

An electronic search was processed on Pubmed, Embase, and Cochrane library for randomized controlled trials (RCTs) comparing the efficacy of diacerein with placebo on T2DM. The primary outcome was fasting blood glucose (FBG). Trial sequential analysis (TSA) was used to test the reliability of this pooled outcome. Secondary outcomes were glycosylated hemoglobin A1c (HbA1c), body mass index (BMI), lipid profiles, hematological indexes including hematocrit and platelet count, and systematic inflammatory level expressed as a C-reactive protein (CRP) level. Safety outcome was the rate of complications. The difference in continuous data was measured by mean difference (MD) and 95% confidence interval (CI), while the difference of dichotomous data was calculated by relative risk (RR) and 95% CI. A two-tailed *P* < 0.05 was regarded as statistically significant.

**Results:**

Five RCTs with 278 participants were included. Compared with control, diacerein provided significant improvement on FBG (MD -0.52; 95% CI (-0.89~-0.14); *P* < 0.05 was regarded as statistically significant. *P* < 0.05 was regarded as statistically significant. *P* < 0.05 was regarded as statistically significant. *P* < 0.05 was regarded as statistically significant. *P* < 0.05 was regarded as statistically significant.

**Conclusion:**

Based on the current analysis, diacerein as an add-on treatment provided better glycemic control for T2DM but this benefit requires more verification. Compared with control, additional diacerein also lowered body weight and CRP level in T2DM, but increased the rate of gastrointestinal syndromes.

## 1. Introduction

Type 2 diabetic mellitus (T2DM) is a metabolic disorder which affects 425 million people in the world, acting as a huge threat on human health as well as an economic burden on the whole society [1]. Uncontrolled blood glucose leads to severe complications including cardiovascular disease, diabetic kidney disease, diabetic retinopathy, and diabetic neuropathy [2–5].

The mechanism of T2DM is complicated. Seeing T2DM as an inflammatory disease is important in understanding the development of the disease. Proinflammatory cytokines (tumor necrosis factor- (TNF-) *α*, interleukin- (IL-) 1*β*, and IL-6) and chemokines are elevated in patients with T2DM [6, 7]. One of the main underlying mechanisms of T2DM is insulin resistance [8]. Increasing evidences indicate that chronic low-grade inflammation plays a vital role in the pathogenesis of insulin resistance. Excessive proinflammatory cytokines stimulate several inflammatory signals such as NF-*κ*B and JNK pathways, resulting in interference with insulin signaling [9]. Given the link between inflammation and pathogenesis of T2DM, anti-inflammation treatment may be beneficial.

Diacerein is an anthraquinone for osteoarthritis treatment through rhein (4,5-dihydroxy-9,10-dioxo-9,10- dihydroanthracene-2-carboxylic acid), which is the active metabolite of diacerein [10]. The main mechanism of diacerein is inhibiting the downstream signaling of IL-1 [10], which is also a major proinflammatory cytokine in the development of T2DM as mentioned above. Diacerein is also effective in inhibiting IL-6 and TNF-*α* signaling [11, 12]. In light of its definite anti-inflammation effect, the potentiality of diacerein on T2DM is increasingly investigated. Animal studies revealed that diacerein reversed the inflammation, decreased fasting glucose, and improved insulin sensitivity [13, 14].

Currently, several randomized clinical trials (RCTs) observed the effect of diacerein on T2DM [15–18]. Given the controversial outcomes across studies, we undertook this systematic review and meta-analysis to summarize the effect of diacerein on T2DM.

## 2. Materials and Methods

This systematic review was organized based on recommended guidelines and in line with the PRISMA (Preferred Reporting Items for Systematic Reviews and Meta-analyses) checklist [19].

### 2.1. Search Strategy

The first two authors independently searched in September 2019 on Pubmed, Embase, and Cochrane library. The keywords used were diacerein and (diabet^∗^). Reference lists of relevant published researches were also reviewed for potentially relevant studies.

### 2.2. Inclusion Criteria and Study Selection

RCTs comparing the effect of diacerein with control (placebo) on T2DM were included. Specifically, patients with comorbidities other than T2DM-related complications were excluded. The first two authors independently reviewed all titles and abstracts for relevance. If the information to make a decision was not enough, full text would be retrieved for further judgment. The senior author would make the final judgment when a consensus could not be reached by the first two authors.

### 2.3. Data Extraction

The same authors independently extracted data from eligible studies including the name of the first author and publication year, location and duration, sample size, inclusion criteria, study arms and participants, and cotreatments. Fasting blood glucose (FBG) was entitled as the primary outcome, in line with other studies [20–22]. Secondary outcomes were glycosylated hemoglobin A1c (HbA1c); body mass index (BMI); lipid profiles including triglyceride (TG), total cholesterol (TC), low-density lipoprotein (LDL), and high-density lipoprotein (HDL); hematological indexes including hematocrit and platelet count; and systematic inflammatory level expressed as the C-reactive protein (CRP) level. Safety outcome was the rate of complications.

### 2.4. Data Analysis

A random-effects model was used for all comparisons because disease duration, detailed intervention methods, and other confounding factors were not consistent across trials [23, 24]. The difference in continuous data was measured by mean difference (MD) and 95% confidence interval (CI), while the difference of dichotomous data was calculated by relative risk (RR) and 95% CI. If the standard deviation (SD) of the change was not reported, a correlation of 0.5 was used to estimate the dispersion [23]. Heterogeneity was assessed by *Q* statistic and *I*^2^ statistic. An *I*^2^ statistic > 50% was considered to have significant heterogeneity [25]. By omitting one study in each turn in a comparison to locate the potential source of heterogeneity, sensitivity analysis was conducted when significant heterogeneity was observed in primary outcome. Publication bias was unable to detect given the limited number of studies included [26]. A two-tail *P* < 0.05 was considered statistically significant. Analyses were conducted by Review Manager, version 5.3 (The Nordic Cochrane Centre, The Cochrane Collaboration; Copenhagen, Denmark).

### 2.5. Quality Assessment

The first two reviewers independently used the Cochrane's risk of bias tool to evaluate the quality of each included study [27]. A value of low, unclear, or high risk of bias was assigned to the following items: random sequence generation, allocation concealment, blinding of participants and personnel, blinding of outcome assessment, incomplete outcome data, selective reporting, and other bias. Disagreement was resolved by discussion. The degree of agreement between two reviewers was quantified by the *κ* value. A *κ* from 0.40 to 0.59 was entitled as fair, 0.60 to 0.74 as good, and 0.75 or more as excellent [27].

The overall quality of evidence for primary outcome was rated according to the Grading of Recommendations Assessment, Development, and Evaluation (GRADE) approach. The level of evidence of pooled results was rated as high, moderate, low, or very low, according to five domains including high risk of bias, imprecision, indirectness, heterogeneity, and publication bias [28–32]. Specifically, publication bias could not be assessed, and evidence was downgraded if heterogeneity was larger than 40% [30].

### 2.6. Trial Sequential Analysis

Traditional cumulative meta-analyses may increase the risk of type I error due to sparse data and repeated significance testing [33]. To control this potential risk, trial sequential analysis (TSA), which was based on the sample size of meta-analysis, was conducted (TSA software version 0.9 Beta; Copenhagen Trial Unit, Copenhagen, Denmark). To make full use of the input data, the empirical method was used for the estimation of required information size. TSA was performed for primary outcome. A diversity-adjusted required information size (DIS) with the eventual breach of the cumulative *Z* curve of the relevant trial sequential monitoring boundaries was obtained so as to acquire the required information size and a threshold for the treatment effect with statistical significance [34]. An overall 5% risk of a type I error was maintained with a power of 80% [34].

## 3. Results

**Figure 1:**
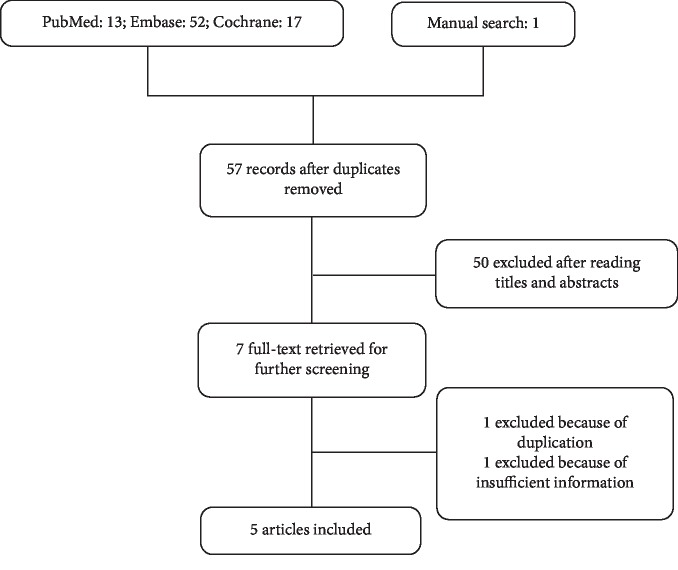
Flow chart.

The initial search yielded 57 titles after deletion of duplication. The full-text versions of 7 studies were retrieved for further screening. One was excluded because of duplication [16] while one was ruled out because of insufficient data [35] (Figure 1). Finally, five RCTs were included [15–18, 36].

### 3.1. Basic Characteristics of Included Studies

A total of 278 patients were included. A 100 mg dose was used in 3 RCTs [16, 18, 36]. Gradient dosages (50 mg and 100 mg) were used in 2 RCTs [15, 17]. Placebo was delivered to participants in the control group in all of the five RCTs. All the participants were instructed to adhere to their antidiabetic recommendations. Patients had to take metformin as monotherapy (≥1500 mg per day) for at least the previous 90 days in one RCT [15]. Patients had received angiotensin-converting enzyme inhibitors or angiotensin II receptor blockers for at least 3 months in one RCT [18]. Therapy regimen was not detailed in the other three RCTs [16, 17, 36]. Basic characteristics of the five RCTs are summarized in Table 1.

**Figure 2:**
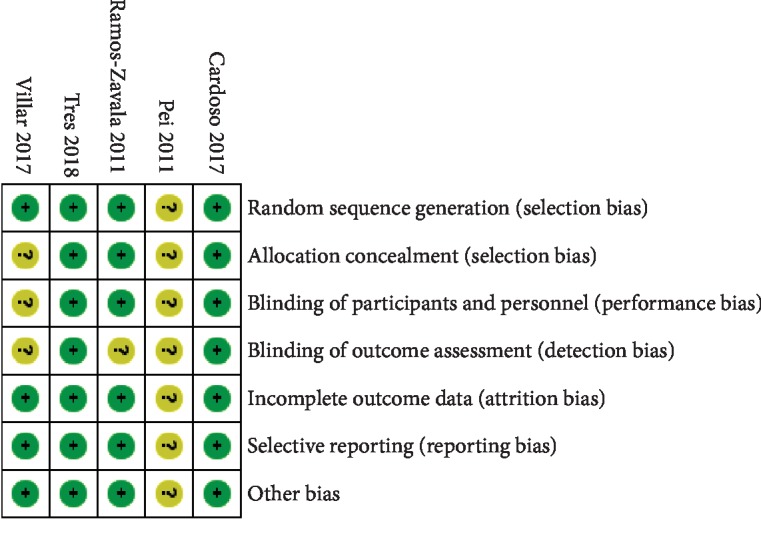
Risk of bias of included studies.

The risk of bias of included RCTs is shown in Figure 2. One study was only a published abstract with limited information for judgment [18]. One study did not mention the detail of allocation and blindness of patients [15], while two did not clarify the blinding of outcome assessment [15, 17]. The *κ* value was 0.873, implying an excellent agreement between two reviewers.

### 3.2. Primary Outcome

**Figure 3:**
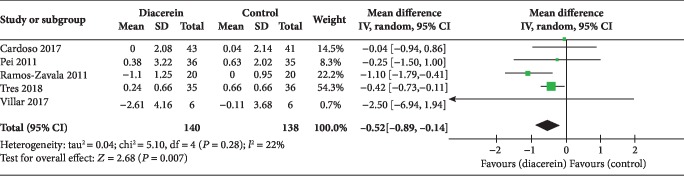
Difference in fasting blood glucose between diacerein and control.

**Figure 4:**
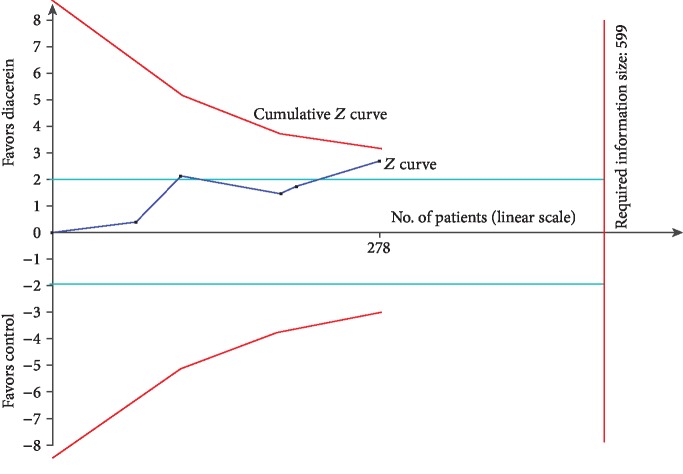
Trial sequential analysis of the pooled outcome regarding fasting blood glucose. A trial sequential analysis (TSA) showed that the pooled results (*Z* curve; blue lines) crossed the conventional boundary of benefit (celeste line) but did not reach the required information size (599) or cross the trial sequential monitoring boundary for benefit (cumulative *Z* curve in red) to enter the area of benefit (above the upper red curve).

All five studies reported the improvement of fasting blood glucose [15–18, 36]. Compared with patients in the control group, diacerein provided improvement of FBG (MD -0.52; 95% CI (-0.89~-0.14); *P* = 0.007) with insignificant heterogeneity (*I*^2^ = 22%, *P* = 0.28) (Figure 3). The level of evidence was moderate. However, this positive effect was questioned by TSA, which suggested that more studies were needed (Figure 4).

### 3.3. Secondary Outcome

The summary of secondary outcomes is listed in Table 2, with all forest plots in Supplemental file ([Supplementary-material supplementary-material-1]). Five studies reported the change of HbA1c [15–18, 36]. Compared with control, patients with supplemental diacerein gained significantly more reduction in HbA1c (MD -0.71; 95% CI (-1.07~-0.36); *P* < 0.001) with significant heterogeneity (*I*^2^ = 78%, *P* = 0.001).

Four studies [15–17, 36] reported results of lipid profiles, and the pooled data showed no superiority in favor of diacerein. Two studies reported hematological indices (hematocrit and platelet count) and inflammatory marker (CRP) [16, 36]. In terms of hematocrit and platelet count, the pooled results showed no priority in favor of either intervention with no significant heterogeneity. On the other hand, the pooled results supported the use of diacerein in a declining CRP level (MD -1.49; 95% CI (-2.78~-0.19); *P* = 0.02) with no significant heterogeneity (*I*^2^ = 0%, *P* = 0.64). Two studies reported data of the change in BMI [15, 36]. The pooled result identified a significant benefit of diacerein (MD -0.40; 95% CI (-0.49~-0.31); *P* < 0.001) without significant heterogeneity (*I*^2^ = 0%, *P* = 0.87).

### 3.4. Safety Outcome

The summary of safety outcomes is listed in Table 3, with all forest plots in Supplemental file. Four studies reported complications [15–17, 36]. The most commonly reported complication was gastrointestinal symptoms including diarrhea, nausea, and abdominal pain. According to the pooled outcome of four studies, compared with control, gastrointestinal symptoms occurred more frequently in patients administered with diacerein (RR 1.39; 95% CI (1.08~1.77); *P* = 0.009) without significant heterogeneity (*I*^2^ = 0%, *P* = 0.85). Two studies reported headache [16, 17], one reported dizziness [16], and one categorized the left complications into other complications [36]. The pooled result showed no superiority in favor of either treatment (Table 3).

## 4. Discussion

This is a further meta-analysis focusing on the effect of diacerein on T2DM as an add-on treatment. According to our results, compared with control treatment, diacerein could benefit glycemic control, as shown by improved FBG and HbA1c, and lower systematic inflammation and BMI, albeit with a similar effect on lipid profiles and hematological indices. On the other hand, diacerein caused more gastrointestinal syndrome, including diarrhea and nausea/vomiting. It should kept in mind that the positive outcome of diacerein over control on FBG needed more verification, as indicated by TSA.

A previous meta-analysis on the same topic reported that diacerein significantly reduced FBG and HbA1c [37]. However, other metabolic indices were not analyzed. In our study, a systematic analysis was made on glycemic control, lipid profile, systematic inflammation, and hematological indexes, displaying a more concrete picture of diacerein on T2DM. Moreover, TSA was used to test the reliability of our meta-analysis, providing guidance for clinicians.

Blood glucose fluctuates widely with intake of food during the day; thus, FBG is used to assess daily average of the level of blood glucose [38–40]. Overnight FBG was commonly used to diagnose DM and to estimate glycemic control [38, 39, 41]. After pooling all studies into analysis, we found additional diacerein as an effective agent in glycemic control, even though this significance requires more confirmation. Meanwhile, glycemic controlling should be evaluated from multisides; none of the single index could depict a full view of glycemic profiles. We further analyzed HbA1c, which was a reflection of an average glucose level over the past three or four months [42]. In accordance with the outcome of FBG, we found that diacerein also reduced HbA1c effectively, suggesting its long-active and steady effect.

Dyslipidemia is one of the main comorbidities associated with diabetes [43]. Abnormal lipid metabolism not only plays a role in development of diabetes but also contributes to the high risk of diabetes-related cardiovascular complications [44]. Current guidelines recommend a tight government of lipid to prevent arteriosclerotic cardiovascular diseases [45]. Combined results of available RCTs showed that diacerein did not favor lipid profiles including TG, TC, LDL, and HDL in comparison with the control group. This suggests that the benefit of diacerein might be independent of influencing lipid profiles.

Previous studies reported that hematological parameters such as red blood cell count, hematocrit, and platelet count are independently related to diabetes [46]. Hematocrit and platelet count were strongly associated with insulin resistance [47, 48]. However, pooled results from our study showed that diacerein has no effect on either hematocrit or platelet count. Given the limited information on insulin resistance (i.e., HOMA-IR) reported by RCTs included, we could not know directly the effect of diacerein on insulin resistance. Evidence from the blood also gives clue to the evaluation of systematic inflammation. CRP is an inflammation-related protein and elevates in T2DM population [49]. We found that diacerein reduced the CRP level significantly, supporting its anti-inflammatory effect.

Evidences show that obesity management is beneficial for the patients with T2DM [50, 51]. In overweight or obese patients with T2DM, moderate weight loss may help control blood glucose and even cut off the demand for hypoglycemic medications [52, 53]. Interestingly, we found that diacerein reduced BMI significantly. On the other hand, additional diacerein caused more gastrointestinal syndrome, among which diarrhea and nausea/vomiting were most frequently reported. Whether this gastrointestinal disorder was related to the decrease of BMI was unclear. For other complications, there was no difference between the diacerein group and control group, supporting the safety of the diacerein.

The strengths of our study resided in meticulous and overall analysis related with diabetes. Our findings will no doubt add confidence to anti-inflammatory therapies for T2DM. However, our study also had several limitations. First, due to the limited number of RCTs, the included population was relatively small, introducing uncertainty to the pooled outcome as shown by TSA. Further clinical trials with larger population were needed. Besides, a comprehensive view of the management of T2DM relies on multifaceted measurements including insulin resistance, fasting and postprandial glucose, and systematic inflammatory markers such as neutrophil lymphocyte ratio. However, there was only one RCT with the assessment of the effect of diacerein on postprandial glucose and even no reports regarding insulin resistance, veiling the systematic understanding of diacerein. Finally, the administration strategies vary widely. Different dosage and durations of treatment may contribute to a heterogeneity.

## 5. Conclusion

Based on the current analysis, diacerein as an add-on treatment provided better glycemic control for T2DM but this benefit requires more verification. Compared with control, additional diacerein also lowered body weight and CRP level in T2DM, but increased the rate of gastrointestinal syndromes.

## Figures and Tables

**Table 1:** Basic characteristics of eligible studies.

Study	Location, duration	Sample size	Inclusion criteria	Study arms and participants	Cotreatment
Ramos-Zavala 2011 [17]	Mexico, 2 months	60	T2DM; age 40-60 years; <6months since diagnosis; overweight or obese (BMI 25.0-34.9); FBG 7.0-11.0 mmol/l; HbA1c 7-9%	T: diacerein (50 mg once daily during the first 15 days and twice daily for 45 additional days) F/M: 12/8; age: 47.5 ± 5.3 C: placebo, F/M: 9/11; age: 47.8 ± 5.2	Usual lifestyle; general recommendations of medical therapy

Cardoso 2017 [16]	Brazil, 48 weeks	84	T2DM; age < 75 years; HbA1c 7.5-9.5%, on either stable oral or insulin treatment	T: diacerein 100 mg/day; F/M: 33/10; age 65.8 ± 6.3 C: placebo; F/M: 33/8; age: 63.7 ± 7.9	Stable oral or insulin treatment

Villar 2017 [15]	Mexico, 90 days	12	T2DM; HbA1c ≥ 7%; age 30-60 years; overweight or obese (BMI 25.0-34.9), with metformin as monotherapy (≥1500 mg per day) for at least the previous 90 days	T: 50 mg of diacerein before breakfast for 15 days and for the remaining 75 days dose was titrated to receive 50 mg of diacerein before breakfast and dinner; F/M: 2/4; age: 41.3 ± 9.7 C: placebo; F/M: 1/5; age: 54.0 ± 3.5;	Metformin as monotherapy

Tres 2018 [36]	Brazil, 12weeks	71	T2DM; age 34-79; HbA1c 7.4-11.0%; no kidney disease;	T:100 mg of diacerein per day; F/M: 15/20; age: 62 ± 8 C: placebo; F/M: 9/27; age 59 ± 11.3	Prescribed glucose-lowering agents and dietary recommendations

Pei 2011 [18]	China, 24weeks	71	T2DM; UACR 50-300 mg/g	T: 100 mg of diacerein per day; F/M: 24/12 C: placebo; F/M: 24/11	ACEi and/or ARB

T2DM: type 2 diabetic mellitus; BMI: body mass index; FBG: fasting blood glucose; T: treatment; C: control; F: female; M: male; ACEi: angiotensin-converting enzyme inhibitor; ARB: angiotensin II receptor blockers.

**Table 2:** Difference in secondary outcomes between the diacerein group and control group.

Index	Number of studies	Sample size of diacerein group	Sample size of control group	Mean difference (95% CI)	*P* value	Heterogeneity (*I*^2^, *P*)
HbA1c	5	140	138	-0.71 (-1.07, -0.36)	<0.001	78%, 0.001
Triglyceride	4	104	103	-7.84 (-18.14, 2.47)	0.14	10%, 0.34
Total cholesterol	3	61	62	-2.83 (-14.92, 9.26)	0.65	41%, 0.19
Low-density lipoprotein	4	104	103	3.91 (-6.53, 14.36)	0.46	69%, 0.02
High-density lipoprotein	4	104	103	-34.6 (-111.11, 41.91)	0.38	100%, <0.001
Body mass index	2	41	42	-0.40 (-0.49, -0.31)	<0.001	0%, 0.87
Hematocrit	2	78	77	0.64 (-0.16, 1.44)	0.12	49%, 0.16
Platelet count	2	78	77	1.00 (-2.09, 4.09)	0.53	0%, 0.62
C-reactive protein	2	78	77	-1.49 (-2.78, -0.19)	0.02	0%, 0.64

**Table 3:** Difference of complications between the diacerein group and control group.

Complications	Number of studies	Sample size of diacerein group	Sample size of control group	Risk ratio (95% CI)	*P* value	Heterogeneity (*I*^2^, *P*)
Gastrointestinal symptoms^∗^	4	104	103	1.39 (1.08~1.77)	0.009	0%, 0.85
Headache	2	63	61	1.25 (0.52~3.01)	0.61	0%, 0.86
Dizziness	1	43	41	1.43 (0.25~8.13)	0.69	Not available
Others^∗∗^	1	35	36	11.31 (0.65~197.11)	0.10	Not available

^∗^Gastrointestinal symptoms include diarrhea, nausea, vomiting, and abdominal pain. ^∗∗^The categories of complications were not specified in the original article.

## Supplementary Materials

Supplementary Materials The forest plots of all secondary outcomes and safety outcomes are listed in supplemental file.
