# Supplementary material for: The Effect of Diacerein on Type 2 Diabetic Mellitus: A Systematic Review and Meta-Analysis of Randomized Controlled Trials with Trial Sequential Analysis
Source: J Diabetes Res. 2020 Feb 10;2020:2593792. doi: 10.1155/2020/2593792 (PMC7035565; doi:10.1155/2020/2593792)

Supplemental Figure 1. Difference in HbA1c between diacerein and control group
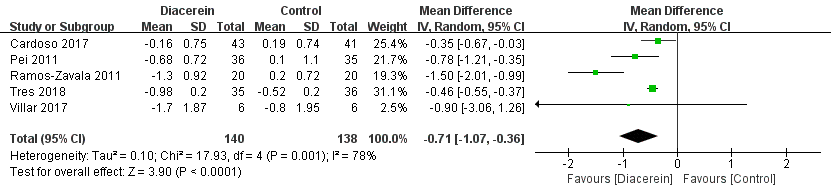


Supplemental Figure 2. Difference in triglyceride between diacerein and control group
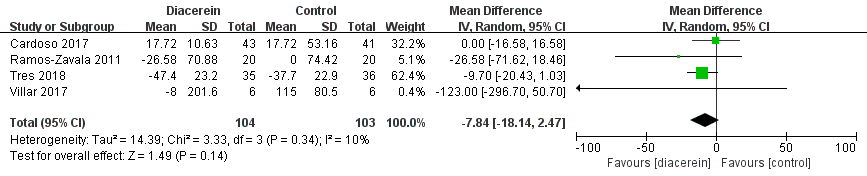


Supplemental Figure 3. Difference in total cholesterol between diacerein and control group


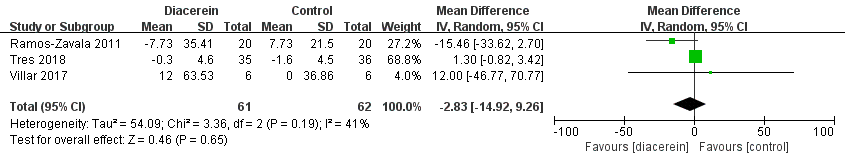


Supplemental Figure 4 Difference in low density lipoprotein between diacerein and control group


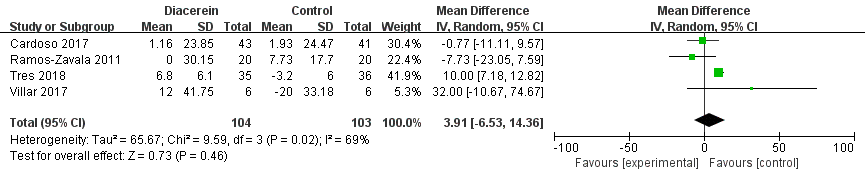


Supplemental Figure 5. Difference in high density lipoprotein between diacerein and control group


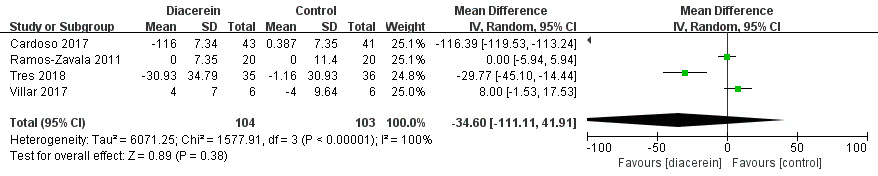


Supplemental Figure 6. Difference in body mass index between diacerein and control group


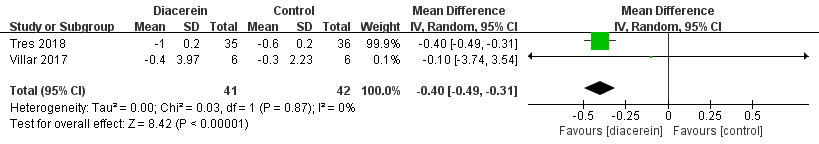


Supplemental Figure 7. Difference in hematocrit between diacerein and control group


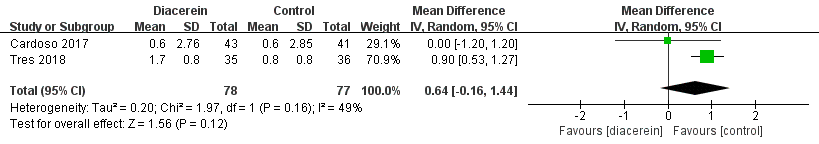


Supplemental Figure 8. Difference in platelet count between diacerein and control group


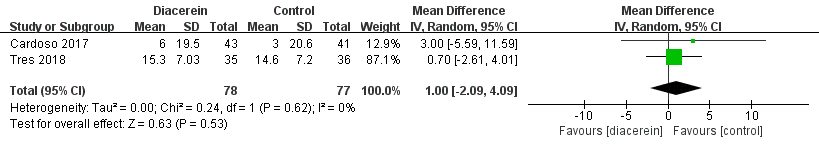


Supplemental Figure 9. Difference in C-reactive protein between diacerein and control group


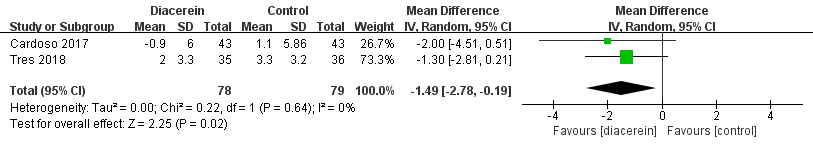


Supplemental Figure 10. Difference in rate of gastrointestinal symptoms between diacerein and control group


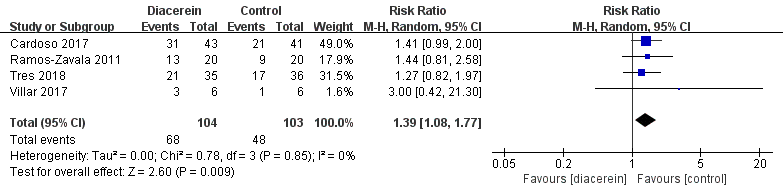


Supplemental Figure 11. Difference in rate of headache between diacerein and control group


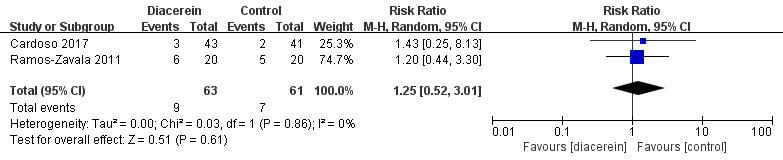

Supplement: Supplementary Materials — The forest plots of all secondary outcomes and safety outcomes are listed in supplemental file. [file 2593792.f1.docx]
